# A Comparison of Emotional Triggers for Eating in Men and Women with Obesity

**DOI:** 10.3390/nu14194144

**Published:** 2022-10-06

**Authors:** Eva Guerrero-Hreins, Lauren Stammers, Lisa Wong, Robyn M. Brown, Priya Sumithran

**Affiliations:** 1 Department of Biochemistry and Pharmacology, University of Melbourne, Parkville 3010, Australia; eguerrerohre@student.unimelb.edu.au; 2 Florey Institute of Neuroscience and Mental Health, University of Melbourne, Parkville 3010, Australia; 3 Department of Medicine, Melbourne Medical School, University of Melbourne, Parkville 3010, Australia; laurenastammers@gmail.com (L.S.); lisa.yuenling.wong@gmail.com (L.W.); 4 Department of Medicine (St. Vincent’s), University of Melbourne, Fitzroy 3065, Australia; 5 Department of Endocrinology, Austin Health, Heidelberg 3084, Australia

**Keywords:** emotional eating, eating behaviour, obesity, gender differences

## Abstract

Objective: Emotional eating (EE) is prevalent in people seeking obesity treatment and is a contributor to poor weight loss outcomes. We aimed to delineate the emotions most associated with this type of eating, and whether they differ by sex in people undergoing obesity treatment. Methods: A cross-sectional study recruiting 387 adults from a hospital obesity management service. Emotional eating was measured using the Emotional Eating Scale (EES). Separate analyses included all participants, and those undergoing lifestyle interventions alone or in combination with obesity medication and/or bariatric surgery. Results: A total of 387 people (71% women) participated in the study (*n* = 187 receiving lifestyle modification alone; *n* = 200 in combination with additional treatments). Feeling ‘bored’ was most commonly and most strongly associated with the urge to eat, regardless of sex or treatment. Women had higher scores for total EES, for subscales of depression and anger, and individual feelings of ‘blue’, ‘sad’ and ‘upset’ compared to men. Conclusions: Understanding why certain emotions differentially trigger an urge to eat in men and women, and finding strategies to break the link between boredom and eating may enable better personalisation of lifestyle interventions for people with obesity.

## 1. Introduction

Current efforts to treat obesity have, for the most part, focused on inducing a negative energy balance and modifying homeostatic drivers of eating (reducing hunger and/or promoting satiation) with pharmaceutical agents and bariatric surgery. Yet, non-homeostatic eating, such as eating in response to environmental or cognitive factors, is a prominent driver of food intake. Non-homeostatic eating behaviours are associated with poorer weight loss after bariatric surgery [1] and behavioural interventions [2], and weight-regain after weight loss [3].

Emotional eating—the tendency to eat in response to positive and/or negative emotions—is present in more than half of patients seeking specialist obesity treatment [4] and is particularly prevalent in women [5]. Women are also more likely to experience mood-related disorders and eating disorders [6]. Reported sex differences in how different emotions affect food intake are largely anecdotal and have not yet been studied empirically. Understanding emotional triggers for eating in men and women is key to developing tailored interventions for people seeking obesity treatment, of whom a large majority are women.

We have previously reported that emotional eating affects 58% of participants attending a public hospital specialist obesity treatment service in Australia [4]. The aims of the current analysis were to characterise the most common emotion(s) prompting an urge to eat, and to examine whether they differ according to sex.

## 2. Methods

### 2.1. Study Design and Participants

This single-centre, cross-sectional study was completed at a tertiary referral hospital in Melbourne, Australia, within a specialist multidisciplinary obesity treatment service. A detailed description of the study design has previously been reported [4]. Briefly, a total of 387 patients attending the obesity treatment service between December 2018 and April 2019 completed the Emotional Eating Scale (EES) [7] over the phone or in-person. Participants were at various stages of treatment. All participants provided written informed consent in accordance with the Declaration of Helsinki and were told their results would not affect their treatment. The study was approved by the Austin Health Human Research Ethics Committee.

### 2.2. Outcome Measures

Demographic information was collected from electronic medical records or directly from participants and is reported elsewhere [4]. Data obtained included age, sex, height, weight, current obesity treatment(s) and history of bariatric surgery.

The EES measures an individual’s urge to eat in the presence of positive and negative emotions [7]. It consists of a 25-item self-reported questionnaire. Each item assesses the desire to eat with a 5-point Likert scale (0 = “no desire” to 4 = “an overwhelming urge to eat”) in the presence of a particular emotion. Scores from each item are summed to obtain a total score out of 100. Item scores are also grouped into three subscales: depression, anger and anxiety. As there is no clinically defined diagnosis for emotional eating, it was classified here with a score threshold of ≥25 (see [4] for details). Our population EES scores had high internal consistency, with a Cronbach’s alpha of 0.95, irrespective of sex.

### 2.3. Statistical Analysis

Normality testing was calculated with the Shapiro–Wilkinson test. Scores of each emotion, and subscale scores, are presented as mode (most common score, individual emotions only), median and interquartile range (IQR). A Friedman’s test evaluated differences between individual emotion scores across all participants. Post-hoc analysis using Dunn’s post-test, and corrected for multiple comparison testing, was done between emotions that had a median score higher than the median split (i.e., emotions with a median score > 2). Mann–Whitney U tests were used to investigate sex differences on median subscale scores and individual emotion scores > 2. To separate the effect of treatment-related factors on sex differences and emotions, analyses were done including all participants (*n* = 387) and including only participants who were (*n* = 200), or were not (*n* = 187), using medication for obesity management or had a history of bariatric surgery at the time they completed the EES. Age (categorised as 20–40, 41–60, 61–79 years old) and body mass index (BMI) (categorised as 28–39, 40–50, 51–75 kg/m^2^) were included as categorical covariates in all analyses. All analyses were performed using IBM SPSS Statistics for Mac, Version 28.0. Armonk, NY, USA: IBM Corp. statistical software.

## 3. Results

### 3.1. Participant Characteristics

Of 504 consecutive clinic patients invited to participate in the study, 78 (16%) declined to participate and 39 (8%) were excluded (see [4] for further information on participant characteristics), leaving 387 participants (187 treated with lifestyle modification only, 200 prescribed additional obesity medication and/or had previous bariatric surgery). The majority (71%) of participants were women; median age was 52 (IQR: 42–61) years and BMI 42 (IQR: 37–49) kg/m^2^.

### 3.2. Most Common Emotion Triggering Overeating

**Figure 1:**
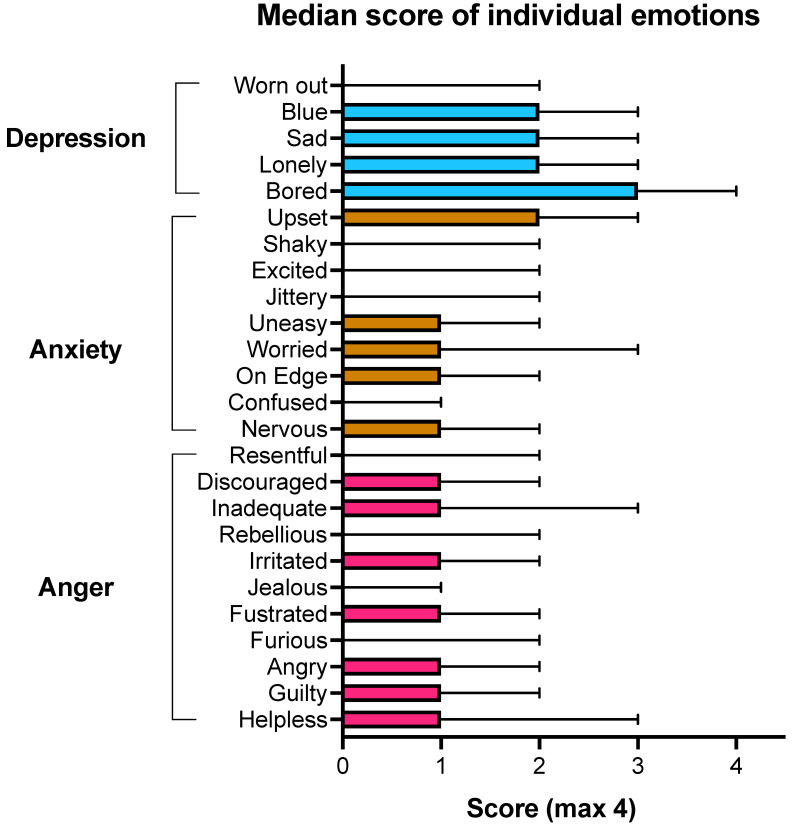
Median and IQR scores of individual emotions from the EES, grouped by subscale for all participants (*n* = 387). Subscales are depression (blue), anxiety (orange), anger (pink).

Across the whole participant group, the emotion most commonly and most strongly identified as prompting an urge to eat was feeling bored (mode = 4; median = 3; IQR: 1–4). The median score for feeling bored was higher compared to all other emotions, except for blue (*p* < 0.0001). ‘Bored’ was the highest scoring emotion in all treatment groups, with no significant differences between groups (Supplementary Table S1). Median and IQR scores for each individual emotion across all participants are shown in Figure 1.

### 3.3. Sex Differences in Emotional Triggers

Emotional eating scores differed by sex, with women having a higher overall median total score of 34 (IQR: 15–49) compared to men (median 25 (IQR: 12–37)). Women also had higher median scores for subscales of depression and anger compared to men (Table 1).

For emotions with a median score higher than the median split, there were no significant differences between men and women for scores on experiencing an urge to eat in response to feeling bored (*p* = 0.21) or lonely (*p* = 0.38). Women reported higher scores for blue (*p* < 0.001), sad (*p* = 0.008) and upset (*p* = 0.01) compared to men.

### 3.4. Sex Differences between Obesity Treatment Groups

Among participants using obesity medication or who had previous bariatric surgery (*n* = 200) there were sex differences in depression and anger subscale scores, and emotions blue and upset, with women reporting higher score responses compared to men (Supplementary Table S1). No sex differences were detected in participants treated with lifestyle intervention alone (*n* = 187) (Supplementary Table S1).

## 4. Discussion

In this cross-sectional study of patients attending a specialist obesity treatment service, the most common emotion identified by participants as triggering an urge to eat was boredom, regardless of sex or type of obesity treatment. Women had higher emotional eating scores overall, particularly for subscales of depression, anger and individual emotions of upset, blue and sad compared to men.

These findings add to the growing literature linking boredom and food intake (including binge eating triggers and food cravings) in people regardless of BMI, age or sex [8,9,10,11,12,13,14,15,16,17]. The present study is the largest investigating specific emotional triggers for eating in adults being treated for obesity and, moreover, the only study to examine emotional triggers by both sex and treatment factors. Previous literature employing the original EES [18], revised EES [15] and children’s version of the EES [19,20] has also shown sex and/or gender differences in depression-subscales. This suggests that while both men and women are susceptible to eating when feeling bored, higher total emotional eating scores in women [4,15,18] may be driven by negative depression-associated emotions.

We also found higher subscale scores for women in anger, but not anxiety. Previous studies using similar subscales are conflicting, showing higher scores for women in anger [19], anxiety [21] and both [20]. Of note, these earlier studies comprised a younger population (children, adolescents or young adults) and employed variations of the EES, such as the children’s EES [20] and Turkish EES, which does not have an anger subscale [21]. Emotional eating in children and adolescents may present itself distinctly from adult emotional eating, given differences in emotional regulation ability and cognitive flexibility [22].

Sex differences in disordered eating behaviour has been previously reported (reviewed in [6]). The reasons are not fully understood. Our study found a depression-specific phenotype for emotional eating in women more than men. Similarly to binge eating and people with type II alcohol use disorder, where women are overrepresented, emotional eating in response to negative affect may be a means to ‘self-medicate’ depressive symptoms. The impact of gender roles will also contribute to observed differences. For instance, the conceptualisation of individual emotions by men and women may differ. A study found ‘worried’ and ‘bored’ were factorially variant (i.e., each item did not measure its intended construct equally across genders) in adolescent boys and girls [23], suggesting the EES could be measuring distinct emotional concepts in women and men when it comes to negative affect. In other words, what men and women categorise as feeling sad or angry could potentially differ, and hence, the perception of frequency they feel those emotions could differ as well. The conceptualisation of emotions in the context of negative emotional eating for men and women requires further research.

The original EES classifies ‘bored’ within the depression subscale. Indeed, boredom and depression have been shown to be highly correlated [24] however, eating out of boredom is a universal experience not necessarily associated with negative wellbeing [25], whereas depression-triggered eating is related to lower psychological well-being [15]. Koball and colleagues revised the EES after finding ‘bored’ was the highest scoring emotion in a group of normal weight female college students to include 6 new items related to boredom, such as feeling restless or unstimulated, and regrouping the emotions under subscales of depression, anxiety/anger and boredom [26]. Studies employing the revised EES support our findings that women have higher scores for wanting to eat in response to depression as well as boredom [14,15]. Few studies which employ the EES report scores for individual emotions. One study reported bored was the most endorsed emotion in a group of children regardless of BMI [27], followed by excited and worn-out. ‘Excited’ was also the most frequent emotion in a group of adolescents, whilst bored was low scoring in this sample [20].

Emotional eating is theorised to be a form of escapism from negative affect [28], which is biologically driven in humans and animals, as consumption of palatable, nutrient-dense foods dampens the stress response [29] and results in release of dopamine, a neurotransmitter associated with reward. Repetitive consumption of palatable foods whilst experiencing negative emotions creates a positive-feedback loop whereby eating as a means to alleviate and distract from negative feelings is reinforced [30]. Greater difficulties in emotion regulation, including the inability to identify and describe emotions, is associated with higher emotional eating [14,15,31,32,33,34]. This is evident for both boredom eating [15,35] and eating in response to negative affect, regardless of BMI [36]. Boredom eating may also be a means to escape aversive self-awareness as a means to pass an otherwise unengaged time [37]. This has been tested in laboratory settings: under boring conditions (e.g., watching boring versus engaging television), participants are more willing to electrocute themselves [13] and consume more food [13,38] regardless of weight or fed state [8]. Importantly, the tendency to experience boredom is predictive of emotional eating [15]. Internal awareness is inversely correlated with binge eating and depression scores [33]. Since overweight individuals have been reported to perceive time to pass more slowly and tend to eat sooner than those of normal weight [39], it may be of value to examine whether targeting emotional regulation and interoceptive awareness in people with obesity who display EE behaviour may improve outcomes of behavioural interventions.

Bariatric surgery or obesity medication status was not significantly associated with any subscale or individual emotion scores. Accumulating evidence suggests bariatric surgery may ameliorate some kinds of disordered eating behaviour such as binge eating [40], and pharmacotherapy such as glucagon-like peptide-1 agonists reduce appetite and may reduce motivation for calorically dense foods [41,42] but less is known about their effects on emotional eating. Interestingly, the relationship between sex and emotional eating was noted only in participants treated with obesity medication and bariatric surgery in addition to lifestyle modification. If confirmed in other studies, examining whether certain obesity treatments have sexually dimorphic effects on emotional eating may be of interest. Notably, the use of medication or history of bariatric surgery did not ameliorate the effects of feeling bored on the drive to eat. This is particularly important as pre-bariatric surgery, feeling bored, stressed and experiencing negative emotions are the highest triggers for overeating [43,44].

This study has several limitations. Firstly, the EES does not include ‘positive’ emotions, with the exception of excited. Nevertheless, eating in response to positive emotions may denote a separate form of emotional eating [45] that does not significantly relate to associated comorbidities such as lower psychological well-being, eating disorder symptoms or poor emotional regulation as negative emotions do [15]. Moreover, whilst not considered an emotion, the scale does not account for feeling stressed, a powerful trigger for overeating, including binge eating [29,46]. Stress is particularly relevant as having greater resources to cope with stress is associated with lower emotional eating scores [47] and variance in overall emotional eating and depression scores is accounted for by perceived stress and stress-coping styles [21,48]. We also did not assess gender identity, which may help delineate whether emotional eating may be more of a psychological, social or cultural phenomenon versus physiological. Observable sex differences in eating disorders may be confounded by the divergent cultural pressures on men and women regarding emotional vulnerability and weight, resulting in underreporting in men [49]. For instance, men may be less likely to disclose sensitive information and/or may be more susceptible to social desirability bias, and have a higher threshold for describing depressive symptoms. Emotional eating may also be more socially acceptable for women and hence more readily reported. The reliance on a self-reported measure, particularly in a treatment setting, introduces some potential bias as people may not accurately report their eating behaviour, thus reducing ecological validity [50,51]. Moreover, this study included participants who were at a specialist obesity clinic seeking weight-loss treatment; thus the findings may not necessarily be generalisable to other populations with obesity who are not actively seeking to lose weight.

## 5. Conclusions

These findings contribute to the growing evidence of sex differences in motivated eating behaviour and highlight the significant role of boredom. Future studies should endeavour to understand how and why specific emotions differentially trigger an urge to eat across people of different genders, and address strategies to break the link between boredom and eating. This may allow the development of personalised strategies for people seeking treatment for obesity.

## Figures and Tables

**Table 1:** Median and IQR scores for emotional eating subscales in women and men.

	Women (*n* = 273)	Men (*n* = 114)	*p*
Total	34 (15–49)	25 (12–37)	0.005
Depression	10 (5–13)	8 (3–11)	0.003
Anxiety	10 (4–16)	8 (4–13)	0.10
Anger	13 (4–16)	9 (2–16)	0.002

*p* values for comparison of median scores between men and women.

## Data Availability

Data will be made available to other researchers upon request.

## Supplementary Materials

The following supporting information can be downloaded at: https://www.mdpi.com/2072-6643/14/19/4144/s1, Table S1: Sex differences in subscale scores in participants treated with lifestyle modification only (*n* = 187) and those with a history of bariatric surgery or current use of obesity medication (*n* = 200).
