# Supplementary material for: A Comparison of Emotional Triggers for Eating in Men and Women with Obesity
Source: Nutrients. 2022 Oct 6;14(19):4144. doi: 10.3390/nu14194144 (PMC9570591; doi:10.3390/nu14194144)
Supplement: Supplementary file 1 [file nutrients-14-04144-s001.zip › nutrients-1895087-supplementary.pdf]

# Supplementary Materials

**Table S1.** Sex differences in subscale scores in participants treated with lifestyle modification only ($n$ = 187) and those with a history of bariatric surgery or current use of obesity medication ($n$ = 200).

| Treatment | Subscale | Males ($n$ = 55) | Females ($n$ = 132) | $p$ |
|---|---|---|---|---|
| Lifestyle management ($n$ = 187) | Depression | 8 (8) | 10 (9) | 0.15 |
| | Anxiety | 9 (9) | 11 (11) | 0.18 |
| | Anger | 9 (12) | 12 (15) | 0.24 |
| | | Males ($n$ = 59) | Females ($n$ = 141) | |
| Bariatric surgery / obesity medication ($n$ = 200) | Depression | 7 (8) | 10 (9) | **.01**** |
| | Anxiety | 8 (11) | 9 (13) | .21 |
| | Anger | 7 (16) | 14 (17) | **<0.001***** |

Values are median (IQR). Maximum possible score for each subscale was 20 for depression, 36 for anxiety and 44 for anger. Mann Whitney U tests were used to calculate p-values.
